# Application of Federated Learning in Cardiology: Key Challenges and Potential Solutions

**DOI:** 10.1016/j.mcpdig.2024.09.005

**Published:** 2024-10-11

**Authors:** Md Saifur Rahman, Chandan Karmarkar, Sheikh Mohammed Shariful Islam

**Affiliations:** a School of Information Technology, Deakin University, Melbourne, Victoria, Australia; b Institute for Physical Activity and Nutrition, Deakin University, Melbourne, Victoria, Australia

Cardiology has been at the forefront of technological innovations. Recent applications of the internet of things (IoT) in cardiology have gained attention for remote patient monitoring and data analysis.^1^ With the surge in IoT data collection, distributed learning has emerged, addressing the challenge of processing vast IoT data sets.^2^ Distributed learning removes computational constraints and enhances response times, reducing reliance on centralized servers and making it easy for cardiology clinics and hospitals to use IoT technologies. However, sharing data among various stakeholders in distributed learning raises privacy concerns. To mitigate this, federated learning has been introduced as a privacy-preserving solution.^3^ In federated learning systems, stakeholders share trained prediction model weights instead of raw data, protecting sensitive information while enabling collaborative model training across organizations. This innovative approach strikes a balance between data analytic efficiency and preserving patient privacy in distributed IoT ecosystems. In this article, we present the application of federated learning in cardiology along with its challenges and discuss potential solutions to overcome these challenges.

Federated learning comes in 2 main types: centralized and decentralized. In centralized federated learning, all stakeholders send their local model weights to a central server, which then aggregates them to create a global model weight. On the contrary, in decentralized federated learning, each stakeholder directly shares its local model weights with other stakeholders to collectively construct a global model. However, centralized federated learning encounters challenges related to its dependency on a single server, affecting its scalability. Decentralized peer-to-peer federated learning has emerged to counter this limitation, mitigating server dependency and resource constraints. In decentralized federated learning, individual stakeholder act as aggregators, collaboratively contributing to creating a global model, illustrated in the Figure. This decentralized federated learning approach fosters greater scalability and resilience, distributing computational load and facilitating collaborative model training across multiple nodes in the network. However, a decentralized federated learning system increases transmission overhead as all clients need to upload their weight to make a global model.

**Figure:**
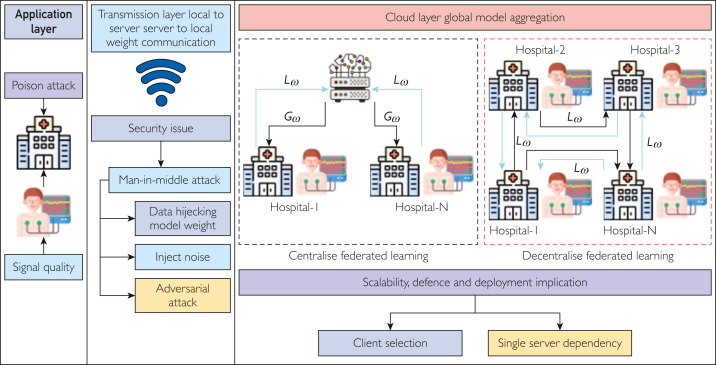
The application of federated learning in cardiovascular disease detection system, where *L*_ω_ is the local model weight and Gω the global model weight.

Federated learning presents a new opportunity in cardiovascular disease (CVD) detection.^4^ This innovative approach enables collaborative model training across diverse health care institutions without the need for centralized data aggregation. By pooling data from various sources, such as hospitals, clinics, and research centers, federated learning enables the creation of robust predictive models for early CVD detection. These models can analyze a wide array of patient data, including electrocardiograms, medical histories, genetic profiles, and lifestyle factors, enhancing diagnostic accuracy. Federated learning allows the development of personalized risk stratification models by integrating diverse patient characteristics and health records. These models can assess an individual’s risk of developing CVD based on unique factors, facilitating targeted preventive interventions and personalized treatment plans.^5^ The decentralized nature of federated learning enables continuous model refinement and adaptation. As new data from various sources becomes available, these models can evolve and improve without sharing raw data, ensuring up-to-date and accurate CVD detection capabilities. Aggregating insights from federated models across various demographic characteristics and geographic regions can offer valuable population-level CVD trends and risk factors. This information can guide public health policies, interventions, and resource allocation for better disease management and prevention.^6^

### Key Challenges

Several challenges exist in using federated learning for automated CVD detection within the internet of medical things framework. Addressing these potential threats is imperative to enhance the acceptability and practicality of federated learning in real-world applications. The key challenges are described further.

#### Application Layer

CVD can be identified through physiologic signals such as electrocardiograms and photoplethysmogram collected from patients. The rise of wearable devices and remote monitoring systems has made physiologic signal collection more accessible. Within federated learning, hospitals and patients serve as clients, leveraging these signals to train models to predict CVDs. However, there are 2 challenging tasks for remote acquisition of physiologic signals for CVD detection as follows:

##### Signal quality

Remote monitoring systems are nonmedical settings, and it is difficult to observe patient movement and daily activities to ensure decent quality signals. Federated learning suffers from data heterogeneity, decreasing model performance.^7^^,^^8^ As a result, handling heterogeneity with noisy signals for CVD detection is challenging compared with that of other domains. Owing to low amplitude signals, physiologic signals are overly sensitive and susceptible to noise.

##### Security

The vulnerability to man-in-the-middle (MITM) attacks poses an important challenge. This form of attack occurs when an unauthorized entity intercepts communication between participating devices and nodes during model aggregation. Man-in-the-middle attackers can potentially manipulate or access sensitive information exchanged between devices, compromising the integrity and confidentiality of the shared model.^9^^,^^10^ For example, where multiple hospitals collaborate for federated learning to improve disease prediction models, an attacker could position themselves between the hospitals’ communication channels. The attacker intercepts these transmissions when the hospitals share their model updates for aggregation. During this interception, the attacker might alter the model updates by modifying the parameters, influencing the aggregated model. Subsequently, the compromised model could provide inaccurate or manipulated insights when deployed for further predictions, impacting the quality and reliability of disease prediction or diagnosis.

#### Potential Solution

Myriad strategies exist to enhance signal quality and mitigate the risks of MITM attacks. Conventional physiologic signal enhancement involves using traditional low-pass, high-pass, and band-pass filters tailored to specific cutoff frequencies.^11^ However, the evolution of deep learning approaches, particularly autoencoder-based data denoizing techniques, has found notably superior performance to traditional filtering systems.^12^

Man-in-the-middle attacks can be mitigated by homomorphic encryption as a safeguard for models against potential attackers. This involves the client sharing an encrypted model with a server, exclusively decryptable by the client.^9^^,^^13^ Consequently, even if an attacker gains access to the communication channel, they are unable to decrypt the model parameters to tamper with the model by injecting noise. Nevertheless, homomorphic encryption presents challenges owing to its complex nature and high computational demands. Another approach to address MITM threats is differential privacy, which introduces noise to the model parameters and makes attackers unable to retrieve the actual model specifics.^14^ Unfortunately, this solution compromises the model’s performance as the added noise affects the accuracy and reliability of the model’s predictions.^15^

#### Transmission Layer

The transmission layer is essential in CVD detection in federated learning. Federated learning needs high computation and bandwidth. For real-time response, it is important to respond immediately if abnormalities are detected. As a result, the following problems are encountered.

##### Device Limitations, Communication cost, and Latency

Wearable devices are resource constraints. Thus, managing memory and computational limitations is crucial, especially in remote monitoring scenarios. Balancing the need for accurate monitoring with these constraints becomes pivotal, ensuring effective cardiovascular health monitoring in resource-limited settings. In federated learning, communication cost refers to the overhead incurred in transmitting model updates and aggregated parameters between the central server and participating devices or nodes^16^ and depends on model convergence.^17^ The size and complexity of the trained machine learning model affect communication costs. More extensive models with numerous parameters require more data transmission during updates, increasing communication overhead.^18^ The number of communication rounds or iterations between the central server and participating devices impacts the overall communication cost. More rounds lead to increased communication overhead. The available network bandwidth determines the speed and volume of data that can be transmitted. Limited bandwidth can slow down communication and increase latency.^19^

##### Security

The transmission layer is affected by not only device limitations but also malicious clients in a federated learning system, where data can be manipulated (eg, data hijacking, injecting noise, and adversarial attacks) to reduce the model performance.^20^

##### Solution

Leveraging edge computing in federated learning can mitigate resource-constrained device problems. Wearable devices transmit data to edge servers to create a preliminary global model before forwarding it to the central server.^21^ This approach optimizes computational resources by offloading initial processing to edge servers, aiding in efficient model aggregation while easing the burden on resource-constrained wearable devices. Communication costs can be reduced by controlling model parameter sizes such as knowledge distillation, model pruning, quantization, and client selection.^18^^,^^22^^,^^23^ Knowledge distillation involves a large model predicting CVD and transferring its learning to a small model, is often referred to as the “teacher-student” model. Pruning eliminates insignificant model weights, reducing model size. However, determining which layers to prune poses a challenge, as defining the appropriate number of layers to eliminate requires careful consideration of the model's performance and structural integrity. Client selection is one of the ways to facilitate communication rounds by selecting the most important clients.^24^ These methods curtail data transmission during updates, minimizing communication overhead in federated learning but striking a balance between size reduction and model accuracy. A challenging task is to identify malicious clients in federated learning, anomaly detection methods at aggregator sites examine model weight transmissions.^25^ Another approach involves scrutinizing latent features of the model.^26^ Any alterations in these latent features signal potential data tampering by malicious clients. Anomaly detection mechanisms flag unusual patterns in the transmitted model weights, indicating probable malicious intent. Similarly, changes in latent features representing the underlying data distributions mean possible data manipulation or unauthorized modifications by malicious entities within the federated learning environment. These methods serve as essential safeguards, enabling the detection and response to potential threats from malicious clients and ensuring the integrity and security of federated learning systems.

#### Cloud Layer

Global model aggregation in federated learning is another vulnerable and costly part of federated learning in the cloud layer as follows:

##### Scalability

The scalability challenge in managing multiple models for aggregation poses notable hurdles in detecting CVD. In CVD scenarios, real-time response and early patient treatment can be critical and directly impact patient outcomes. Relying on a single-server-based aggregation method poses substantial risks; a server failure in other domains might be manageable within hours but could be problematic in cardiology. Deploying federated learning for CVD detection necessitates a thorough consideration of server scalability. Additionally, the maintenance expenses of servers could limit medical providers to transition to the global model concept through federated learning.^17^^,^^27^^,^^28^ Balancing the need for scalability with cost concerns becomes imperative while advancing medical approaches reliant on such technology.

##### Stakeholder Selection

Selecting stakeholders for global model aggregation is pivotal in CVD detection systems to enhance model performance. As data heterogeneity grows due to patient variations and signal diversity across sources, excluding less substantial models during selection might lead to model overfitting, posing an elevated risk. The noticeable heterogeneity in physiologic signals worsens this issue, restricting the applicability of federated learning in cardiology.^29^ Achieving a balance in stakeholder selection considering diverse data inputs while mitigating the risk of model overfitting is an important challenge in leveraging federated learning in cardiology.

##### Solution

Decentralized federated learning can solve the scalability and single server dependency.^28^ However, decentralized federated learning increases communication overhead, challenging real-time CVD detection. Stakeholders should be included considering data diversity^30^ in physiologic signals and using appropriate software to manage diverse physiologic signals and contribute to global model improvement. A summary of potential challenges in federated learning is presented in the Table and Figure.

**Table:** Possible Challenges in the Federated Learning–Based Disease Section in the Internet of Medical Things Framework

Attributes	Challenges
Man-in-middle attack	Federated learning creates a global model by sharing the client model. During sharing, attackers can do adversarial attacks to manipulate model parameters.
Communication cost	In each communication round, all or a portion of the client model needs to communicate, which involves bandwidth cost.
Latency	It is essential to train global models in a faster way to predict diseases in real-time. However, not all clients have the same bandwidth.
Non-IID problem	As federated learning uses diverse client data, a data heterogeneity problem exists in optimizing the global model. The non-IID (non-Independent and Identically Distributed) problem refers to the situation where the data distributions across different clients are not uniform or similar.
Malicious client	As federated learning involves multiple clients, there is a chance to join a malicious client who can inject noise into the data to manipulate global model performance.
Computational resources	Edge devices have limited resources to build a model, which is lacking in the deployability model in edge devices.

### Personalized Care

It is essential to track CVD conditions in daily living conditions. However, the pattern of physical signals varies from patient to patient based on the heart condition. Federated learning can create a robust, personalized model to capture variations for early detection of CVD. As a result, it will be easier for clinicians to manage personalized risks and medications for individual patients.

### Hospital Patients

Timely monitoring of CVD in high-risk patients, especially postsurgery, is crucial. However, early detection can be challenging for hospitals as CVD symptoms vary among patients, race, geographical location, and behaviors. Sharing patient data for comprehensive analysis is restricted owing to privacy concerns. Federated learning addresses this challenge by facilitating the sharing of a robust model capable of detecting CVD. This approach enables collaborative learning from diverse hospital patient populations worldwide without compromising individual data privacy. By leveraging the collective knowledge embedded in local data sets, federated learning empowers hospitals to improve CVD detection accuracy, ensuring early identification of diverse health conditions whereas respecting privacy constraints.^17^^,^^31^

### Conclusion

In conclusion, federated learning is ideal for collaborative learning among diverse health care providers while protecting patient privacy. This innovative system facilitates the classification of unknown patterns of CVDs across nations. Despite its privacy-preserving nature, vulnerabilities persist, including potential attacks and challenges in aggregating diverse client data. However, numerous solutions have been proposed to address these concerns. Yet, these solutions necessitate further assessment concerning varying health care domains, demographic perspectives, optimization of models on edge devices and scalability across different settings. Rigorous evaluation and adaptation of these solutions tailored to specific health care contexts are imperative for the seamless implementation and efficacy of federated learning in enhancing cardiac care.

## Potential Competing Interests

Dr Islam is funded by the 10.13039/501100001030National Heart Foundation of Australia (102112) and 10.13039/501100000925National Health and Medical Research Council Emerging Leadership Fellowship (APP1195406). The other authors report no competing interests.
